# Obeticholic acid and 5β-cholanic acid 3 exhibit anti-tumor effects on liver cancer through CXCL16/CXCR6 pathway

**DOI:** 10.3389/fimmu.2022.1095915

**Published:** 2022-12-20

**Authors:** Haoxian Gou, Shenglu Liu, Linxin Liu, Ming Luo, Shu Qin, Kai He, Xiaoli Yang

**Affiliations:** ^1^ Department of Hepatobiliary Surgery, The Affiliated Hospital of Southwest Medical University, Luzhou, China; ^2^ Academician Workstation of Sichuan Province, Luzhou, China

**Keywords:** single cell RNA sequencing, hepatocellular carcinoma, immunotherapy, CXCL16, natural killer T cells, bile acids

## Abstract

Hepatocellular carcinoma (HCC) is the most common type of liver malignancy with a high incidence and mortality rate. Previous *in vitro* and *in vivo* studies have confirmed that liver sinusoidal endothelial cells (LSEC) secrete CXCL16, which acts as a messenger to increase the hepatic accumulation of CXCR6^+^ natural killer T (NKT) cells and exert potent antitumor effects. However, evidence for this process in humans is lacking and its clinical significance is still unclear. In this study, by dissecting the human HCC single-cell RNA-seq data, we verified this process through cellphoneDB. NKT cells in patients with high expression of CXCL16 exhibited a higher activation state and produced more interferon-γ (IFN-γ) compared with those with low expression. We next investigated the signaling pathways between activated (CD69 high) and unactivated NKT cells (CD69 low) using NKT cell-developmental trajectories and functional enrichment analyses. *In vivo* experiments, we found that farnesoid X receptor agonist (obeticholic acid) combined with the takeda G protein coupled receptor 5 antagonist (5β-cholanic acid 3) exhibited significant tumor suppressive effects in the orthotopic liver tumor model and this result may be related to the CXCL16/CXCR6 axis. In conclusion, our study provides the basis and potential strategies for HCC immunotherapy based on NKT cells.

## 1 Introduction

Hepatocellular carcinoma (HCC) is one of the most prevalent human cancers associated with a high mortality rate (1). Due to the low resection rate of HCC and the high recurrence risk after hepatectomy or transplantation, comprehensive treatments including surgery, interventional therapy, targeted treatment, and emerging immunotherapy approaches are necessary (2). Immune checkpoint inhibitors, such as nivolumab and pembrolizumab, which target programmed cell death protein-1 (PD1) mainly enhance the T-cell response to tumors, thus improving the prognoses of patients with HCC (3, 4). However, only approximately 20% of patients with malignancy benefit from this approach (5).

In addition to T cells, the liver is also abundant in natural killer T (NKT) cells which account for 25% of human liver lymphocytes and 40% of mouse liver lymphocytes (6). NKT cells are a heterogeneous subpopulation of T cells coexpressing T-cell receptors and various receptors that are abundantly expressed in NK cells (7). Activated NKT cells exert powerful antitumor function through multiple mechanisms involved in the granzyme and perforin pathway, release immunomodulatory cytokines including interferon (IFN-γ) and tumor necrosis factor (TNF), and form the Fas/FasL and TRAIL/TRAILR complex (8–10). Therefore, immunotherapy based on targeting NKT cells is a promising and novel strategy to improve the outcomes in patients with HCC.

CXCL16, a chemokine mainly secreted by liver sinusoidal endothelial cells (LSEC) in the liver triggers the hepatic recruitment of CXCR6^+^ NKT cells, thus exerting a potent antitumor effect (11, 12). Ma, et al. found that CXCL16 not only promotes the recruitment of NKT cells to the liver but also promotes the activation of intrahepatic NKT cells, as manifested by the elevation of some activation markers (such as CD69) and the increase of antitumor cytokine (such as IFN-γ) (13). Zhu, et al. also found that CXCR6 deficiency impaired the IFN-γ producing capacity of hepatic NKT cells, and downgraded the accumulation of NKT cells (14). The gut microbiome mediates the conversion of primary bile acids to secondary bile acids. Primary and secondary bile acids have different effects on CXCL16 production in LSEC. Specifically, primary bile acids upregulate CXCL16 secretion in LSEC, enhancing antitumor activity, whereas secondary bile acids exhibit the opposite response (13, 15). Although these findings provide new targets for the treatment of HCC, controlling the gut microbiome or directly altering the composition and ratio of primary and secondary bile acids is complicated.

**Figure 1:**
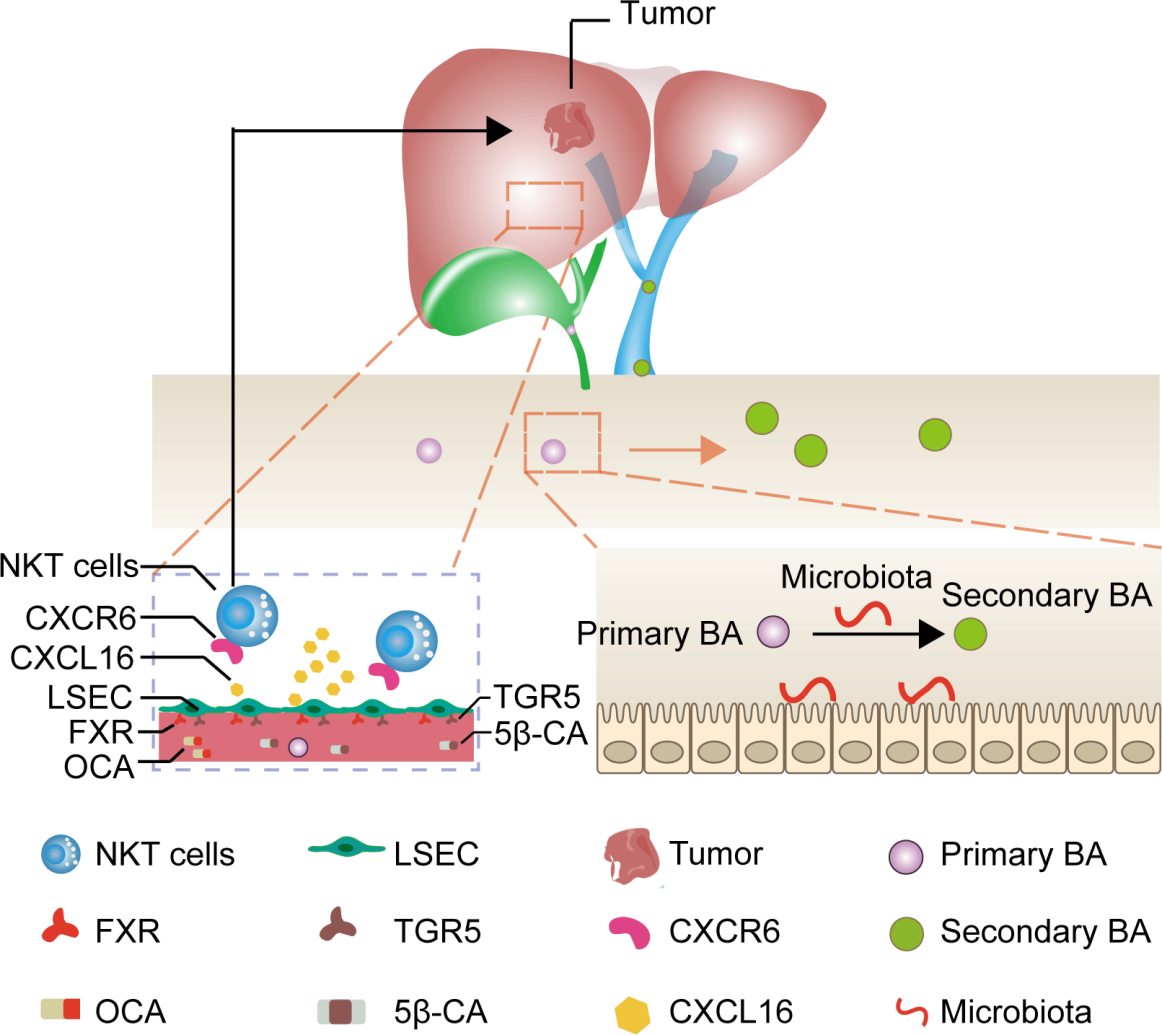
Mechanism of liver cancer immunotherapy mediated by OCA and 5β-CA. OCA binds to FXR and 5β-CA binds to TGR5, thus upregulating CXCL16 secretion from LSEC. The increase in CXCL16 promotes NKT cell accumulation in the liver and tumor-suppressive effects.

Primary bile acids are predominantly bound to the farnesoid X receptor (FXR) and secondary bile acids are primarily bound to the takeda G protein coupled receptor 5 (TGR5) in LSEC (16–18). Therefore, we assumed that applying drugs binding to both FXR and TGR5 could directly target CXCL16 production in LSEC, bypassing the bile acids as well as microbiota metabolism, thereby being a promising, feasible immunotherapeutic approach to treat HCC. Given this background, we evaluated the FXR agonist obeticholic acid (OCA) to mimic primary bile acids and 5β-cholanic acid 3 (5β-CA) to block the downstream signaling of secondary bile acids as an approach to treating HCC using an orthotopic HCC mouse model (**Figure 1**).

Single-cell RNA sequencing (scRNA-seq) is an efficient method for investigating cell heterogeneity, cell-cell interactions, and cell evolution. Given that the interaction of LSEC with NKT cells has not been validated using tissue samples of patients with HCC, we dissected 53,982 human single-cell transcriptomes to explore the feasibility of targeting the LSEC-CXCL16/CXCR6+ NKT cells axis in humans.

## 2 Materials and methods

### 2.1 scRNA-seq data collection

The GSE149614 dataset was obtained from the GEO database, which included 71,915 cells from 10 patients with HCC and from 4 relevant sites: primary liver tumor, normal liver, metastatic lymph node, and portal vein tumor thrombus. Among them, 8 patients with HCC having primary tumor tissues and paired normal liver tissues were selected for this study. Two other patients were excluded as only tumor samples were available.

### 2.2 Data processing

Cell filtering, normalization, dimensionality reduction, clustering, and cell-type annotation were performed using the “Seurat” package (version 4.1.1) in RStudio. The gene-barcode matrix was filtered to exclude low-quality cells (< 600 or > 7000 detected genes, >15% mitochondrial genes) and low-expressed genes (any gene expressed in < 10 cells). Finally, 53,982 cells from primary tumor tissue (26,259 cells) and normal liver tissue (277,23 cells) were selected for subsequent analysis.

Raw counts data were normalized using the “NormalizeData” function with the default parameters (scale.factor = 10000). The top 1500 most variable genes obtained using the “vst” method were used for principal components analysis (PCA). Principal components (PCs) were identified using the “RunPCA” and “Jackstraw” functions. The first 16 PCs were used for cluster identification and t-Distributed Stochastic Neighbor Embedding (t-SNE) dimensionality reduction with the parameter “res = 0.8.” The marker genes of the generated clusters were determined by running the “FindAllMarkers” function with the parameter “only.pos = TRUE, min.pct = 0.25.” Next, cell-type annotation was based on the “CellMarker” database and the existing literature.

### 2.3 Copy number variation inference

Single cells with clonal large-scale chromosome CNV were evaluated using the “inferCNV” R package (version 1.10.1) with the default setting. The setting parameters included “denoise = T,” “cutoff = 0.1,” and “HMM = F.” T cells and B cells from adjacent liver samples were input as the reference group and other cells as the observation group.

### 2.4 Cell–cell communication analysis

To explore potential receptor–ligand interactions between different cells, the “CellphoneDB” python package (version 3.0.0) was used for cell–cell communication analysis. CellphoneDB is a public repository that includes a known list of ligands, receptors, and their interactions from existing literature.

We investigated the possible presence of ligand–receptor pairs between endothelial cells, myeloid cells, NKT cells, LSEC, hepatocytes, T cells, and B cells in normal liver tissues adjacent to the tumor tissues, and also ligand–receptor pairs between malignant cells, myeloid cells, NKT cells, T cells, and B cells in primary tumor tissues. To quantify the potential communication between these cells, general circos plots and detailed plots for each major cell type were drawn using the igraph package in RStudio. We then selected chemokine ligand–receptor pairs in normal liver tissues and immune-related ligand–receptor couples in primary tumor tissues to draw bubble diagrams.

### 2.5 Grouping of patients based on scRNA-seq data

We calculated the proportion of LSEC to all cell types in tumor samples and paired normal liver samples separately by the “proportions” function in RStudio. Next, we assigned 8 patients and 4 patients in each group. The adjacent liver tissues in all patients had a cell count ≥ 1940 (**Supplementary Figure 4**). Thus, to avoid classification errors arising from the varying total number of cells per patient, we randomly selected 1940 cells of each patient from the normal liver tissue using the “sample” function and subsequently calculated the total amount of CXCL16 expressed by all LSEC in the selected cells and used it as a score for CXCL16 expression in that patient. Lastly, we divided patients into a CXCL16 high-expression group (CH) or a CXCL16 low-expression group (CL) based on their CXCL16 expression scores.

### 2.6 Cell developmental trajectory

To infer the differentiation trajectory of NKT cells, we used the “Monocle” package (version 2.22.0) to conduct pseudo-temporal analysis on CD69high and CD69low NKT cells. We first used the ‘‘FindAllMarkers’’ function to determine the differentially expressed genes (DEGs) between CD69high and CD69low NKT cells. Next, we ordered each NKT cell along the trajectory for pseudo-temporal analysis, drew a heat map based on the expression changes of characteristic differential genes using the “plot_pseudotime_heatmap” function, and divided these characteristic differential genes into 5 clusters based on their expression patterns. Based on Gene Ontology (GO) analysis for the biological process, we further annotated the critical clusters with different expression patterns to functional categories.

### 2.7 DEGs identification and GSEA analysis

We first identified the DEGs between CD69high and CD69low NKT cells using the “FindMarkers” function and mapped the volcano plot and heat map. The “clusterProfiler” package (version 4.2.2) was used for GSEA analysis and the gene sets included the Reactome, Kyoto Encyclopedia of Genes and Genomes (KEGG), and GO (biological process) databases. We applied the “sort” function to rank DEGs from large to small and used the “GSEA” function to acquire results from GSEA. Lastly, GSEA enrichment plots were drawn to show significantly activated and suppressed pathways.

### 2.8 Cell lines and animals

Mouse liver tumor cells (H22) were purchased from PriCells (Wuhan, China). H22 cells were cultured with Roswell Park Memorial Institute-1640 (RPMI-1640) medium containing 1% penicillin-streptomycin and 10% fetal bovine serum (FBS) and incubated at 37°C in an atmosphere of 5% CO2.

The Southwest Medical University Animal Management Committee approved all animal experiments. Seven-week-old male Kunming (KM) mice weighing 18–22 g were purchased from the Experimental Animal Center of Southwest Medical University. All animals were housed in cages with 5 mice/cage and provided access to food and water ad libitum. All cages were maintained at a constant temperature of 20−24°C and relative humidity of 50%.

### 2.9 Modeling and pharmacologic interventions

KM mice were used to establish the H22 orthotopic liver tumor model. Anesthetized male KM mice were fixed on an operation table. A longitudinal midline incision was made to allow complete liver exposure. H22 cells extracted from the ascites fluid of KM mice were washed with sterile saline and diluted to 4×107 cells/mL with phosphate buffer saline (PBS). Next, 0.01 mL of the H22 cell suspension (4×105 cells) was slowly injected into the left lobe of the liver of each mouse. The puncture made by the syringe needle was immediately pressed using a 75% ethanol-dipped cotton swab to kill the extravasated cancer cells and prevent the leakage of tumor cells into the peritoneal cavity. Mice were randomized into 4 groups after 3 days of surgery and treated with different drugs.

OCA and 5β-CA were purchased from Beijing Jianqiang Weiye Technology Co. Ltd. (Beijing, China). To upregulate CXCL16 secretion in LSEC, mice were gavaged with OCA (20 mg/kg), or 5β-CA (20 mg/kg), or OCA+5β-CA (20 mg/kg+20 mg/kg) every 2 days at 3 days after H22 inoculation. Polysorbate-20 (Tween-20) was added to dissolve OCA or 5β-CA, which was then diluted in PBS. Vehicle (Tween-20 and PBS)-gavaged mice served as the controls. At 17 days after cell inoculation, the liver tissues, tumors, serum, and intestinal contents were collected and the weights of the liver and tumor were recorded.

### 2.10 Biochemical and cytokine analysis

To assess the adverse effects of the drugs, serum biochemical parameters including aspartate aminotransferase (AST), alanine aminotransferase (ATL), creatinine (CRE), and blood urea nitrogen (BUN) were analyzed according to the instructions in the corresponding assay kits.

The levels of CXCL16, IFN-γ, and TNF-β in the serum, normal liver tissues, and tumor tissues were determined using the corresponding enzyme-linked immunosorbent assay kits (RUIXIN Biotech, Quanzhou, China) in accordance with the manufacturer’s instructions. We used “pg/g” as the concentration unit of cytokines in the tumor and adjacent normal liver tissues and “pg/mL” as the concentration unit for the serum.

### 2.11 Histological analysis

The collected tumors, and liver, kidney, and lung tissues were fixed with 4% paraformaldehyde, paraffin-embedded, and sectioned to a thickness of 5 μm. Subsequently, the sections were stained with hematoxylin and eosin (H&E), and the tumors as well as pharmacological-induced liver, kidney, and lung damage were visualized using microscopy. Histological photographs were acquired and analyzed using optical microscopy (Leica, Germany).

### 2.12 Analysis of intrahepatic immune cells

Tumor tissues were mechanically clipped, filtered through a 300-mesh filter, and centrifuged, and the cell concentration was adjusted to 106 cells/mL with PBS. Next, 100 μL of cell suspensions were taken in sterile Eppendorf tubes; 1 μg each of CD8a, CD3, CD49b, CD45, CD4, Live/Dead, CD69, and CXCR6 antibodies were added, thoroughly mixed, and stained at 4°C for 30 min while protecting from light. The cells were detected using a ZE5 flow cytometer (Bio-rad, American) and the data were analyzed using Flowjo software.

### 2.13 Statistical analysis

Two-tailed unpaired Student’s t-test was used to calculate statistical differences between groups. Data analyses were performed using RStudio software. P < 0.05 was considered statistically significant (*0.01 ≤ P value < 0.05, **0.001 ≤ P value < 0.01, ***P value < 0.001).

## 3 Results

### 3.1 Determination of cell types in the scRNA-seq dataset

**Figure 2:**
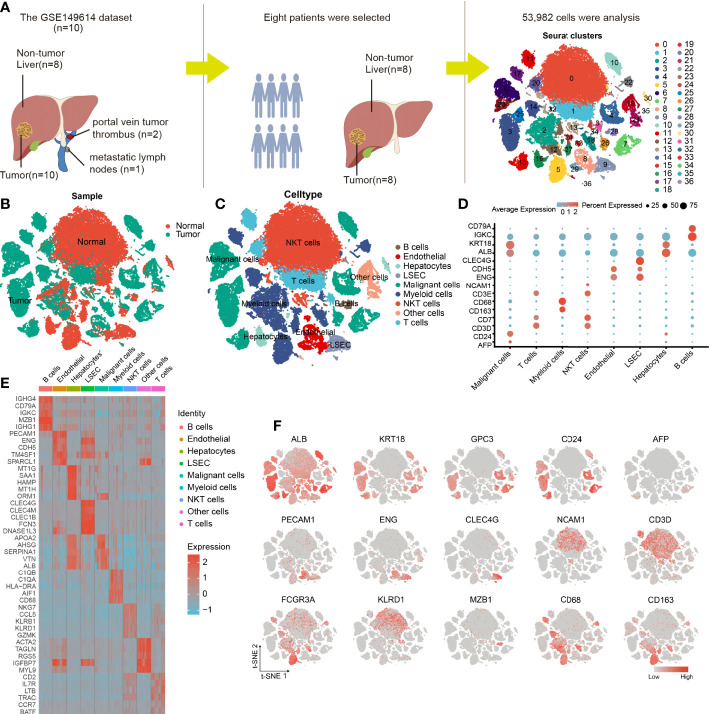
Cellular annotation of scRNA-seq data for tumor and adjacent liver tissue samples from 8 patients. **(A)** Processing procedure of GSE149614 scRNA-seq dataset and t-SNE distribution of 53,982 cells. **(B)** The cluster map illustrates the cell origins by color (non-tumor liver and HCC samples). **(C)** The t-SNE plot shows the 8 identified cell types and 1 undefined cell type (annotated as other cells) from 10× Genomics. **(D)** The bubble plot displaying the normalized expression levels of selected marker genes across the 9 clusters, the variation from high to low expression is represented by the colors from orange to blue. **(E)** Heatmap of top 5 DEGs in the identified cell types. **(F)** Feature t-SNE plots showing the expression levels of 15 well-known marker genes.

Eight tumor specimens and paired adjacent nontumor liver samples were selected from the GSE149614 dataset for subsequent analysis. After the initial quality controls, we retained 53,982 cells for cell annotation (**Supplementary Figures 1**, **2**). Thirty-seven cell clusters from tumor and normal liver tissues were identified and visualized using t-distributed stochastic neighbor embedding (t-SNE) dimensionality reduction (**Figures 2A, B** and **Supplementary Figure 3**).

The cells in clusters 3, 4, 6, 7, 10, 15, 23, 25, 26, and 35 originated from tumor specimens and were highly expressed in ALB, KRT18, and CD24; therefore, they were labeled as malignant cells (**Figures 2C, F**). Furthermore, these malignant HCC cells were also defined by inferred CNV (**Supplementary Figure 3**). We noticed that AFP, an HCC marker, was lowly expressed in liver cancer cells, this finding was consistent with what was reported by Sun et al. (19), suggesting that it may not be a reliable marker gene for HCC. The possible reason is that AFP is not universally expressed in all liver cancers. Similarly, we observed that the cells in cluster 16 and 30 were all derived from non-tumor liver and highly expressed in GPC3 and CD24; thus, they were annotated as hepatocytes (**Figures 2C, F**).

The nonimmune cells that were identified mainly included LSEC (CLEC4G and ENG) (20, 21), non-LSEC endothelial cells (endothelial, CDH5, and ENG), and hepatocytes (ALB and KRT18). The immune cells that were identified consisted of T cells (CD3E and CD7), B cells (CD79A and IGKC), NKT cells (NCAM1 and CD3D), and myeloid cells (CD68 and CD163) (**Figures 2C, D**). Next, we performed differential gene expression analysis among 9 cell types to verify the accuracy of cell annotation (**Figure 2E** and **Supplementary Table 1**). The identified immune cell types were co-present in the tumors and adjacent liver sections but at different proportions, revealing the heterogeneity of the HCC microenvironment. Despite this variation, tumors and adjacent liver specimens had similar proportions of B cells (**Supplementary Figure 4**).

### 3.2 Cell-cell communication in human HCC

**Figure 3:**
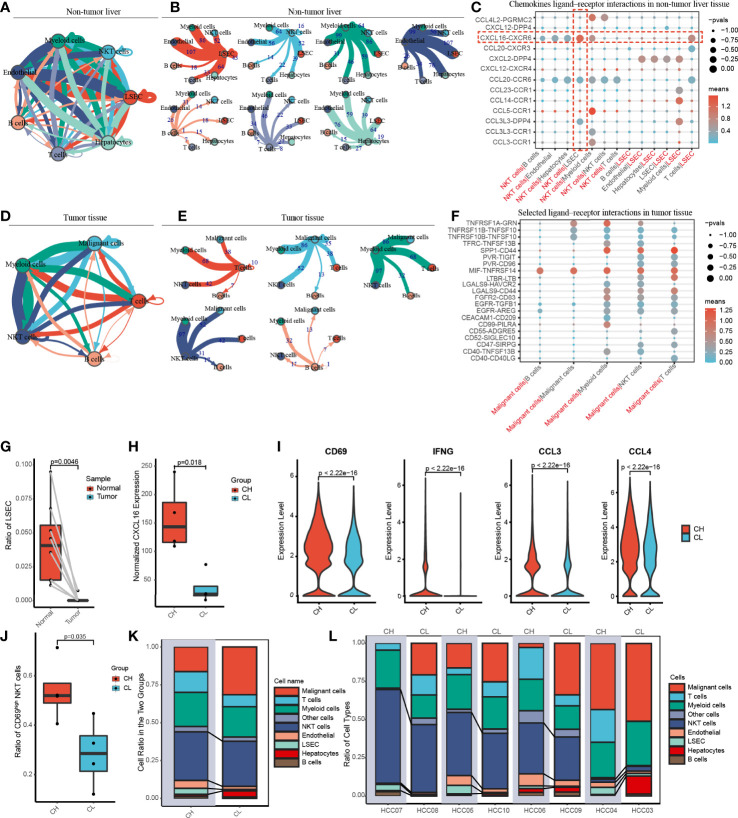
Cell-cell communication networks and patient grouping. **(A, B)** The map showing in detail the number of putative ligand–receptor interactions between each cell type and others in the non-tumor liver. The width of the connecting lines is proportional to the imputed events, which are also labelled with numbers. **(C)** Summary of selected chemokines ligand-receptor interactions that may exist between LSEC and other cells in the noncancerous tissue. The size of the circles is inversely proportional to the p-value, and the color variation represent the level of interaction. **(D, E)** The graph quantifies potential communication between various immune cells and malignant cells in tumor tissue. **(F)** Detailed view of the selected ligand-receptor interactions between major immune cells and cancer cells. **(G)** The ratio of LSEC in tumor and adjacent normal liver samples. **(H)** According to the level of CXCL16 expressed by LSEC, eight patients were divided into the CXCL16 high group (CH) and the CXCL16 low group (CL). **(I)** Violin plots indicating CD69, IFNG (IFN-γ), CCL3 and CCL4 were highly expressed in NKT cells from the CH sample. **(J)** The boxplot displaying the ratio of CD69^high^ NKT cells in CH and CL. **(K)** Overview of the proportion of each cell type in CH and CL. **(L)** The histograms showing the proportion of cell types in each patient.

To gain insight into cell–cell interactions in HCC, we calculated the number of ligand–receptor pairs among tumor tissues and paired nontumor liver tissues using the CellphoneDB. The heat map shows the level of interactions between each cell type and others (**Supplementary Figure 5**), and the number of ligand–receptor pairs is presented by color variation. Myeloid cells were notably active and interacted with all cell types in the liver tissues adjacent to the tumor (**Figures 3A, B**).

Previous animal experiments have found that LSEC recruits CXCR6+ NKT cells by secreting CXCL16. However, whether this mechanism also exists in humans remains unclear. We have widely identified chemokine ligand–receptor complexes between LSEC and other cell types. The bubble diagram revealed that the extent of CXCL16/CXCR6 signaling was the highest between LSEC and NKT cells, suggesting that the mechanism of LSEC using CXCL16 as a messenger to recruit NKT cells may similarly present in human HCC (**Figure 3C**). In addition to NKT cells, LSEC may also recruit T cells *via* the CXCL16/CXCR6 complex. Interestingly, several rarely reported cell–cell interactions (CXCL2/DPP4, CCR1/CCL23, CCR1/CCL14, CCL3L3/DPP4) between LSEC and myeloid cells were identified, suggesting that LSEC may recruit myeloid cells by multiple ways. In addition to LSEC, NKT cells may also recruit myeloid cells through receptor–ligand singling including CCL5/CCR1, CCL3/CCR1, and CCL4L2/PGRMC2 (**Figure 3C**).

In the tumor samples, we noticed that B cells had the weakest interaction with tumor cells, indicating that B cells may have relatively weak antitumor effects (**Figures 3D, E**). We further analyzed cell–cell interaction in tumor tissues and an interesting finding was that TNFRSF14, a co-stimulatory molecule (22), interacted with MIF widely existing in all major immune cell types and malignant cells of the liver, indicating that TNFRSF14/MIF signaling may be a promising target of immunotherapy in HCC (**Figure 3F**).

### 3.3 Patient grouping

We investigated the proportion of LSEC in adjacent liver and tumor tissues and found that LSEC were almost absent in the tumor regions and exclusively restricted to the adjacent liver sections (**Figure 3G** and **Supplementary Figure 4**). In the liver, it is known that CXCL16 is mainly secreted by LSEC. Therefore, based on the total amount of CXCL16 expressed by LSEC in each patient, the 8 patients in our study were categorized into either a CXCL16 high-expression (CH) group or CXCL16 low-expression (CL) group, with 4 patients in each group (**Figure 3H**).

To explore the differences in immune function between both groups, we performed differential gene expression analysis. We noticed that the activation marker CD69, antitumoral cytokine IFN-γ, and chemokines CCL3 and CCL4 were notably highly expressed in NKT cells in the CH group (**Figure 3I**). In addition, samples in the CH group contained a much higher proportion of CD69high NKT cells (**Figure 3J**). These findings suggested that higher expression of CXCL16 is associated with a stronger function of NKT cells. Furthermore, the histograms indicate that the CH group has a higher proportion of NKT cells and LSEC (**Figures 3K, L**). Therefore, in human HCC, CXCL16 may also trigger the hepatic accumulation of NKT cells. This phenomenon was previously identified only in animal models of liver cancer.

### 3.4 Pseudotime trajectory analysis of NKT cells

CD69 is an activation marker of NKT cells. We found that the level of CD69 normalized expression was substantially higher in the CH group compared with that in the CL group; moreover, the CH group had a higher percentage of CD69^high^ NKT cells (**Figures 3I, J**). To determine the correlation between CD69 expression and NKT cell function, we investigated the alterations in gene expression and associated functional variations during the differentiation of CD69^low^ to CD69^high^ NKT cells using pseudotime trajectory analysis.

**Figure 4:**
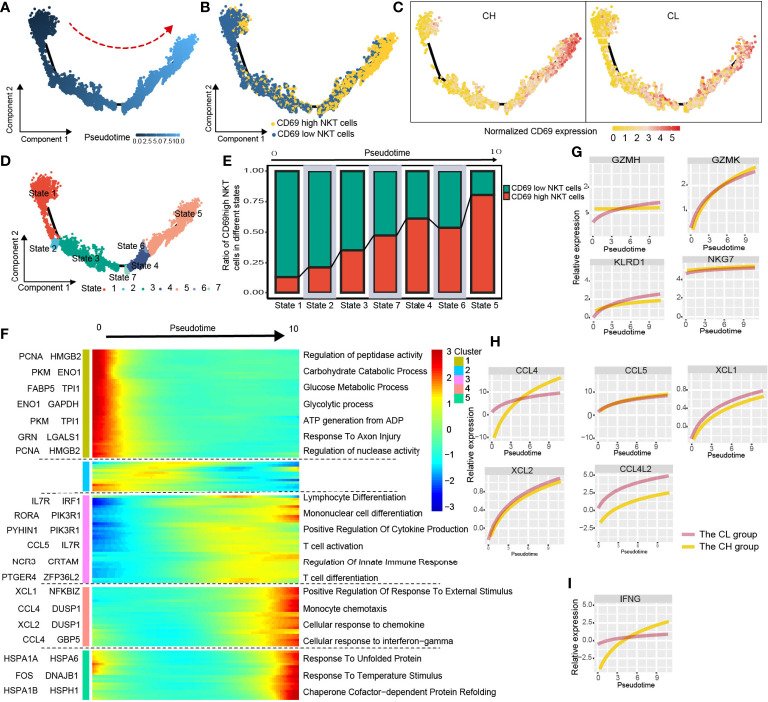
Simulation of NKT-cell differentiation trajectory in HCC. **(A)** The red arrow represents the simulated development direction of NKT cells. **(B)** 2D trajectory chart showing the distribution of CD69^high^ NKT cells and CD69^low^ NKT cells along with the trajectory. **(C)** 2D graph showing the dynamics of normalized expression of CD69, from CH and CL samples. **(D)** All cells are divided into 7 states and labeled with different colors. **(E)** The graph showing the proportion of CD69^high^ NKT cells in different states, along the developmental trajectory. **(F)** Heatmap displaying the dynamic variation of differentiation-related gene expression. The dynamic expression of cytotoxic-related genes **(G)**, chemokines **(H)** and anti-tumoral cytokine IFNG (IFN-γ) **(I)**.

CD69^low^ NKT cells were predominantly distributed at the beginning of the differentiation trajectory, whereas CD69^high^ NKT cells were primarily found at end of the trajectory (**Figures 4A, B**). Next, we analyzed normalized CD69 expression along the trajectory in samples from the CH and CL groups separately. At end of the trajectory, the normalized CD69 expression of NKT cells obtained from patient samples in the CH group was significantly higher compared with that in the CL group (**Figure 4C**). All cells were divided into 7 states along the differentiation process (**Figure 4D**). As the cell state transitioned along the differentiation trajectory, the ratio of CD69^high^ NKT cells increased gradually (**Figure 4E**).

We studied the variation in gene expression patterns associated with NKT cell state transition and determined 149 dynamic genes with notable alterations in expression; these genes were divided into 5 clusters (**Supplementary Table 2**). Next, we extracted these dynamic genes from different clusters for GO analysis. Cluster 1 was characterized by the downregulated expression of metabolism-related genes such as PCNA, HMGB2, PKM, and ENO1. GO analysis revealed that signaling pathways such as the carbohydrate catabolic process, glucose metabolic process, and ATP generation were enriched in cells at the beginning of the trajectory (CD69^low^ NKT cells) (**Figure 4F** and **Supplementary Figure 6A**). Interestingly, clusters 3 and 4 were characterized by the upregulated expression of immune cell differentiation–related genes such as IL7R, IRF1, RORA, and NCR3, and chemokines including CCL4, CCL5, XCL1, and XCL2. Moreover, enrichment analysis indicated that the pathways involved in the differentiation of lymphocytes, mononuclear cells, and T cells; positive regulation of cytokine production; regulation of innate immune response; and cellular response to chemokines and IFN-γ were enriched in cells at end of the trajectory (CD69^high^ NKT cells) (**Figure 4F**, **Supplementary Figure 6B, C**). Additionally, we found that cytotoxicity-related genes including GZMH, GZMK, KLRD1, and NKG7; chemokines including CCL4, CCL5, XCL1, XCL2, and CCL4L2; and the anti-tumoral cytokine IFN-γ were gradually upregulated as differentiation progressed (**Figures 4G-I**). These findings collectively suggested that CD69^high^ NKT cells had stronger immune function compared with CD69^low^ NKT cells but may be at a disadvantage with respect to substance metabolism.

### 3.5 Identification of DEGs and GSEA

**Figure 5:**
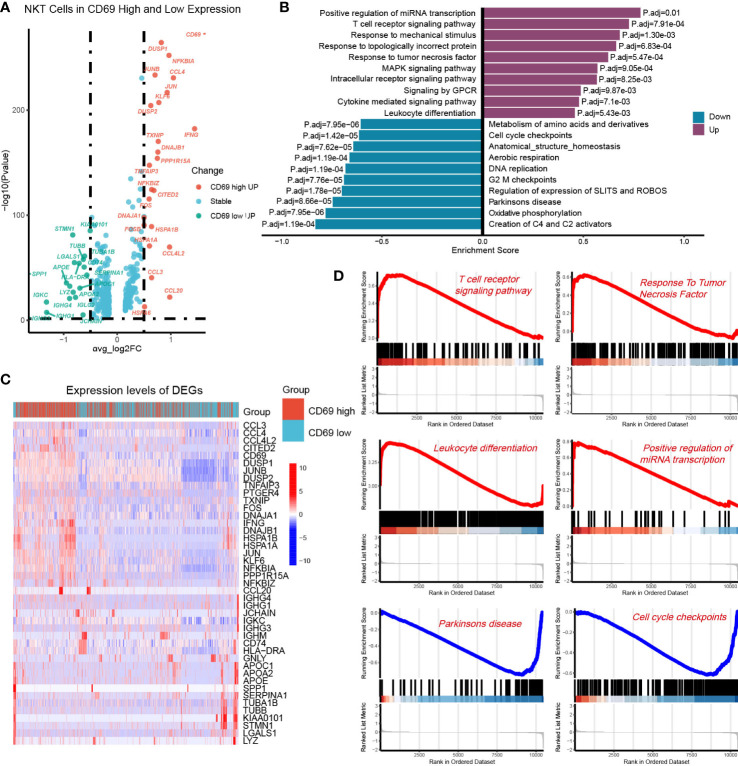
Identification of DEGs between the CD69^high^ NKT cells and CD69^low^ NKT cells. **(A)** Volcano plot showing the DEGs between CD69^high^ NKT cells and CD69^low^ NKT cells. Red and green colors represented high expression genes within CD69^high^ and CD69^low^ NKT cells, respectively. The blue color represented the genes with no significant changes. **(B)** Heatmap displaying the top 43 significant DEGs between CD69^high^ NKT cells and CD69^low^ NKT cells. Red and blue colors represented upregulated and downregulated genes, respectively. **(C, D)** GSEA functional enrichment analysis of the ranked DEGs. The numbers marked on the chart represent the adjusted P-values. The purple and blue colors represented upregulated and downregulated pathways in the CD69^high^ NKT cells, separately. The gene sets included Reactome, KEGG and GO (Biological process) databases.

To further investigate the functional differences between CD69high NKT cells and CD69low NKT cells, we identified their DEGs and performed GSEA. Volcano plots and heat maps displaying the most significant DEGs are depicted in **Figures 5A, B** and **Supplementary Table 3**. We performed GSEA using genes ranked by the absolute value of log2 (fold change). We observed that the pathways associated with the T-cell receptor signaling pathway, response to TNF, and leukocyte differentiation were mainly enriched in CD69high NKT cells, whereas pathways related to the cell cycle such as cell cycle checkpoints were primarily enriched in CD69low NKT cells (**Figures 5C, D**).

### 3.6 OCA combined with 5β-CA suppresses orthotopic H22 liver cancer development

**Figure 6:**
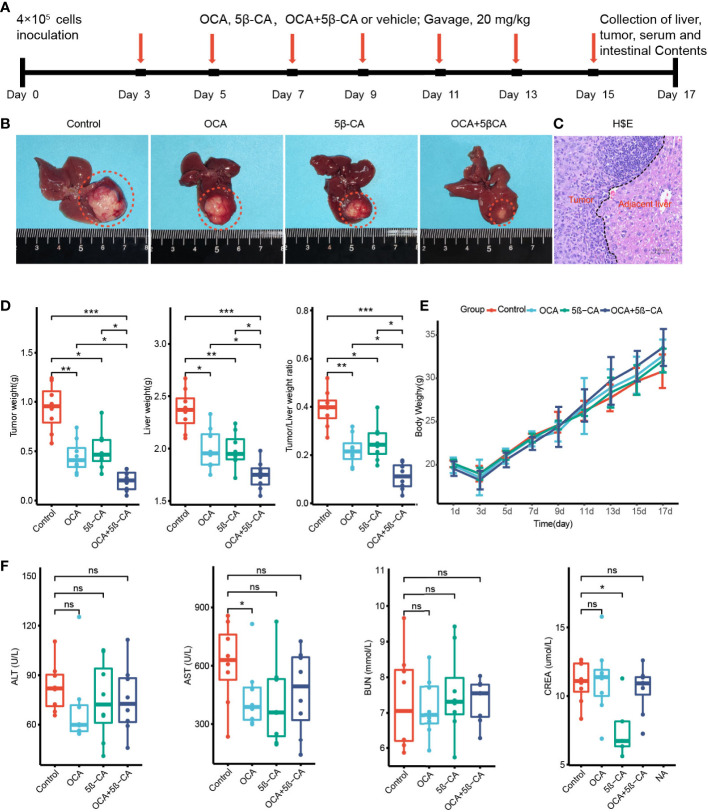
OCA combined with 5β-CA significantly inhibited orthotopic H22 liver tumor. **(A)** The experimental flow chart. **(B)** Representative liver images in the 4 groups are shown. The tumors were outlined with an orange dashed curve. **(C)** The orthotopic H22 mice model was assessed by H&E images. A black dashed line outlines the border between the tumor and the non-tumor liver. **(D)** The liver weight, tumor weight and the ratios of tumor/liver weight from 4 groups were recorded while mice were executed. **(E)** The body weight variation. **(F)** Tumor-induced liver injury, drug-induced hepatotoxicity or drug-induced liver protective effect was assessed by serum levels of ALT and AST. Drug-induced nephrotoxicity was assessed by BUN and CREA (ns, no significance; *0.01 ≤ P value < 0.05; **0.001 ≤ P value < 0.01; ***P value < 0.001).

We constructed an H22 orthotopic liver cancer mouse model to better mimic the immune microenvironment of human HCC. First, mice were inoculated with H22 cells inoculation on day 0 and were then treated with various drugs (Tween-20 and PBS, OCA, 5β-CA, OCA+5β-CA) (**Figure 6A**). At the end of the experiment, representative macroscopy images of livers from the 4 groups are shown in **Figure 6B**. H&E staining was used to evaluate the microscopic features of H22 orthotopic liver tumors. The cancer cells varied in size and exhibited large nuclei and increased mitotic figures, suggesting the successful establishment of the orthotopic model of liver cancer (**Figure 6C**).

Tumors and livers were collected on day 17 to compare the tumor burden between different groups. The tumor and liver weights of the OCA and 5β-CA treatment groups were lower than those of samples obtained from the control group, indicating a partial tumor-suppression effect. Samples from the OCA+5β-CA group had the lowest tumor and liver weights, revealing a more pronounced therapeutic effect. Additionally, the OCA+5β-CA group exhibited the smallest tumor/liver ratio (**Figure 6D**). The body weights were recorded every 2 days to evaluate tumor-induced cachexia. No apparent body weight loss was observed in any group except during the first 3 days after tumor inoculation, indicating that all mice were in the early disease stage (**Figure 6E**). No significant differences in serum ALT and AST levels were determined between mice treated with different drugs, suggesting that OCA and 5β-CA did not exert obvious hepatoprotective effect or hepatotoxicity. OCA and 5β-CA were not associated with nephrotoxicity or pulmonary toxicity as serum BUN and CRE levels were similar among all 4 groups (**Figure 6F** and **Supplementary Figure 6**).

### *3.7 In vivo* immunity analysis after various treatments

**Figure 7:**
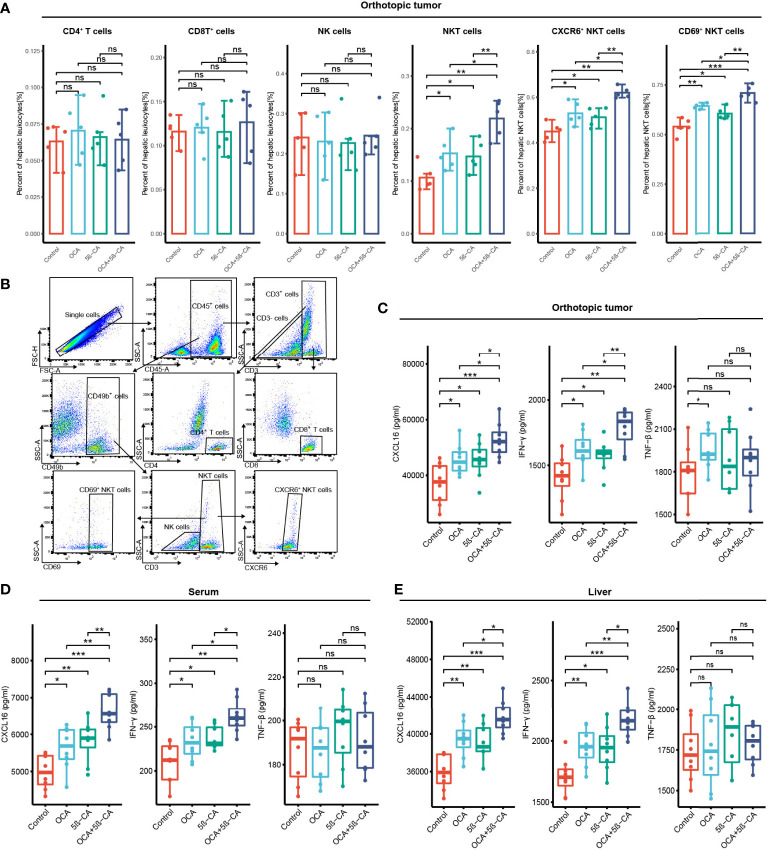
OCA plus 5β-CA improving the number and enhancing the function of NKT cells. **(A)** Proportions of tumor-infiltrating CD4^+^ T cells, CD8^+^ T cells, NK cells, NKT cells, CXCR6^+^ NKT cells and CD69^+^ NKT cells after various treatments. **(B)** Flow cytometry diagrams showing the gating strategy of CD4^+^ T cells (CD3^+^ CD4^+^), CD8^+^ T cells (CD3^+^ CD8^+^), NK cells (CD49b^+^ CD3^+^), NKT cells (CD49b^+^ CD3^+^), CXCR6^+^ NKT cells, CD69^+^ NKT cells. **(C-E)** CXCL16, INF-γ and TNF-β concentrations in the orthotopic tumor, serum and normal liver adjacent to the tumor after various drug treatments (ns, no significance; *0.01 ≤ P value < 0.05; **0.001 ≤ P value < 0.01; ***P value < 0.001).

*In vivo* immunity analysis was conducted to further explore the molecular mechanism of OCA+5β-CA-mediated significant tumor growth inhibition. On day 17, orthotopic tumors were digested into single-cell suspensions for flow cytometry. OCA+5β-CA treatment led to a remarkable increase in NKT cells, whereas CD4^+^ T, CD8^+^ T, and NK cells did not exhibit much change. A slight increase in the number of NKT cells was observed in the OCA and 5β-CA groups (**Figures 7A, B**). To provide mechanistic insight into the increase in NKT cell numbers and to investigate whether OCA+5β-CA treatment affected NKT cell activation, we analyzed the NKT cell phenotype of CXCR6 and CD69 (a marker of NKT cell activation). Notably elevated proportions of CD69^+^ NKT cells and CXCR6^+^ NKT cells were observed after treatment with OCA+5β-CA (**Figures 7A, B**).

INF-γ and TNF-β derived from NKT cells are beneficial for NKT-initiated antitumor immunity. Higher IFN-γ levels in the serum, tumors, and livers were found in NKT cells obtained from OCA+5β-CA-treated mice; however, TNF-β levels did not change obviously (**Figures 7C-E**). CXCL16, a ligand for CXCR6, is mainly secreted by LSEC and recruits CXCR6^+^ NKT cells to the liver. Elevated CXCL16 levels in the tumors, serum, and livers of OCA+5β-CA-treated mice explained the accumulation of intratumoral NKT cells (**Figures 7C-E**). Importantly, OCA+5β-CA exhibited superior effects in enhancing NKT cell infiltration, promoting their activation and, hence, increasing the antitumor function of NKT cells compared with OCA and 5β-CA monotherapy (**Figure 7A-E**).

## 4 Discussion

So far, only immune checkpoint inhibitors targeting the PD1/PDL1 axis are available to clinicians for HCC immunotherapy, and their response rates are still limited (23, 24). NKT cells, with robust antitumor activity, store thousands of fold higher levels of antitumor cytokines than NK cells and CD8^+^ T cells (25–27). Previous *in vitro* and *in vivo* experiments have confirmed that LSEC use CXCL16 as a messenger to control the accumulation of CXCR6+ NKT cells in the liver (11, 13, 14, 28). However, this evidence in human samples is lacking. Based on human scRNA-seq data, we found that high CXCL16 expression in LSEC was associated with an increased number and activated phenotype of NKT cells. Further animal experiments determined that OCA combined with 5β-CA was highly effective in upregulating CXCL16 production, thereby increasing the hepatic number of NKT cells and exerting a significant tumor growth–inhibition effect.

By forming ligand–receptor complexes, cell–cell communication plays a crucial role in coordinating diverse biological processes in the tumor immune microenvironment (29, 30). CellphoneDB is an open database where ligands, receptors, and their interactions are stored, enabling users to achieve a systematic and comprehensive analysis of intercellular communication. Notably, the CellphoneDB takes into account the ligand and receptor subunits and, therefore, allows an accurate representation of the heteromeric complexes (31). We used the CellphoneDB and found that LSEC express relatively high levels of CXCL16, and that CXCR6 (the receptor of CXCL16) is overexpressed in NKT cells, revealing the potential interactions in biological processes between LSEC and NKT cells.

CD69 is a classic activation marker of NKT cells (32). NKT cells exhibit potent antitumor effects upon activation by rapidly releasing multiple cytokines such as IFN-γ, TNF-β, and chemokines (33, 34). Differential analysis suggested that compared with NKT cells from the CL group, those from the CH group were characterized by the overexpression of CD69, IFN-γ, CCL3, and CCL4, suggesting an activated phenotype. In addition, samples from the CH group had a higher proportion of NKT cells. Therefore, hepatic CXCL16 levels correlated with the accumulated number and immune function of NKT cells.

Cells constantly switch from one differentiated state to another throughout the growth cycle. This process has also been described as cell differentiation. Cells in different differentiation states have distinct gene expression patterns, resulting in dynamic cell function changes (35). During cell differentiation, the expression of some genes was gradually reduced while that of others was activated (36). The purification and extraction of cells in different differentiation states are complex, leading to difficulties in describing the gene expression transients in animal or cellular experiments. Pseudotime trajectory is a method that enables the determination of dynamic gene expression patterns and functional status alterations without having to purify cells (37). Results from the analysis indicated that during the differentiation of CD69^low^ NKT to CD69^high^ NKT cells, cytotoxic-related genes such as GZMH, GZMK, KLRD1, and NKG7; chemokines such as CCL4, CCL5, XCL1, XCL2, and CCL4L2; and the classic antitumoral cytokine IFN-γ were gradually upregulated. For GO analysis, we extracted the significantly dynamic genes during the differentiation process of CD69^low^ NKT to CD69^high^ NKT cells. The results suggested that the signaling pathways involved in the differentiation of lymphocytes, mononuclear cells, and T cells; positive regulation of cytokine production; regulation of innate immune responses; and cellular response to chemokines and IFN-γ were significantly enriched. We further determined the DEGs between CD69^high^ and CD69^low^ NKT cells and conducted GSEA. The pathways associated with the T-cell receptor–signaling pathway and the response to TNF and leukocyte differentiation were mainly enriched in CD69^high^ NKT cells. These findings demonstrated that CD69^high^ NKT cells exerted more cytotoxicity and had higher antitumor immune functions compared with CD69^low^ NKT cells.

Pathak et al. (38) reported that the FXR agonists alter the abundance of bile acid–producing bacteria, thus increasing the levels of the secondary bile acid taurolithocholic acid (TLCA) by thousands of times. Based on this finding, we speculated that when OCA was used alone, the increase in TLCA may reduce CXCL16 secreted from LSEC by binding to TGR5, and the TGR5 antagonist may offset this TLCA-induced adverse event. Therefore, we tested the combination of OCA and 5β-CA for the treatment of HCC using an orthotopic HCC mouse model. OCA+5β-CA treatment led to the highest proportion of NKT, CXCR6^+^ NKT, and CD69^+^ NKT cells. NKT cells that accumulated after OCA+5β-CA treatment produced more IFN-γ. In addition, OCA+5β-CA treatment led to the lowest tumor and liver weights compared with that using the vehicle or using OCA or 5β-CA monotherapy, suggesting that the combination treatment may be a promising immunotherapeutic approach in the management of HCC.

## 5 Conclusion

Our study presents the important targets and potential immunotherapeutic strategies for the treatment of HCC. By dissecting the scRNA-seq data, we verified that in the human HCC microenvironment, CXCL16 secreted from LSEC could also increase the accumulated number of hepatic NKT cells. The accumulated NKT cells in the liver in turn exhibited a higher activation state and produced more IFN-γ. We also investigated the differences in gene expression patterns and signaling pathways between activated CD69high NKT cells and unactivated CD69low NKT cells using NKT cell-developmental trajectories, differential analysis, and functional enrichment analysis. *In vivo* experiments in an orthotopic liver tumor mouse model were conducted to confirm the enhanced antitumor efficacy of the combination of OCA with 5β-CA.

## Data availability statement

Publicly available datasets were analyzed in this study. This data can be found here: GSE149614.

## Ethics statement

The animal study was reviewed and approved by Southwest Medical University Animal Ethics Committee.

## Author contributions

HG and SL wrote the original draft. HG, SL and LL performed the animal experiments. ML performed the flow cytometry. SQ prepared the packages on RStudio. KH designed this study. XY supervised the whole experiments and reviewed the original draft. In addition, HG and SL contributed equally for this work. All authors contributed to the article and approved the submitted version.
