# Supplementary material for: Obeticholic acid and 5β-cholanic acid 3 exhibit anti-tumor effects on liver cancer through CXCL16/CXCR6 pathway
Source: Front Immunol. 2022 Dec 20;13:1095915. doi: 10.3389/fimmu.2022.1095915 (PMC9807878; doi:10.3389/fimmu.2022.1095915)
Supplement: Supplementary file 4 [file DataSheet_4.docx]

**Supplementary Figure.1**


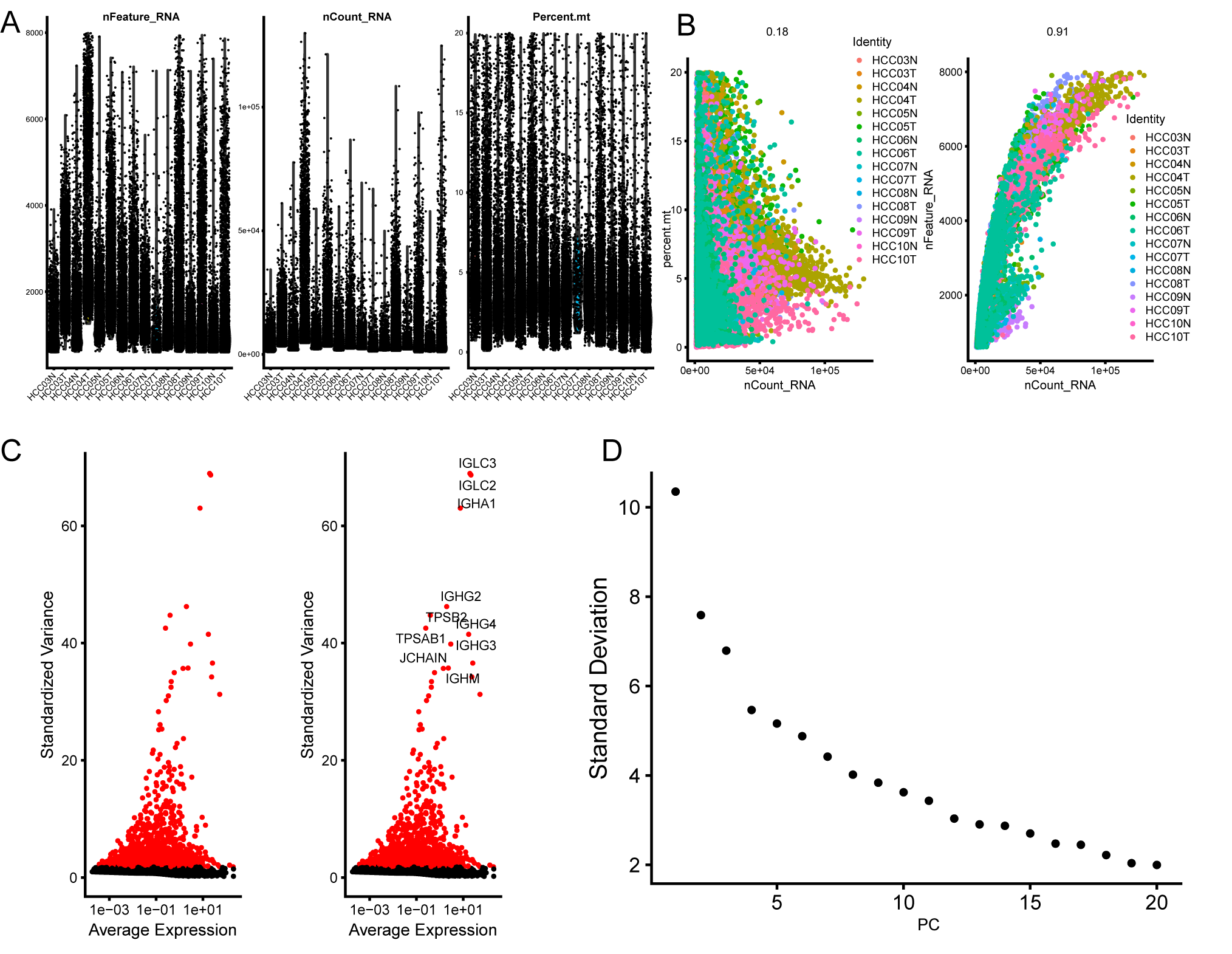


**Supplementary Figure 1: Characterization of single-cell RNA sequencing from**

**human healthy liver and HCC environments.** (A) Quality control of scRNA-seq for 8 HCC patients from liver and tumor samples to filter out the cells with poor quality. (B) We analyzed the correlations between detected gene counts and sequencing depth. (C) We identified the high variable genes across cells and drew the characteristic variance diagram. (D) We drew the elbow plot to determine the optimal PCs number.

**Supplementary Figure.2**


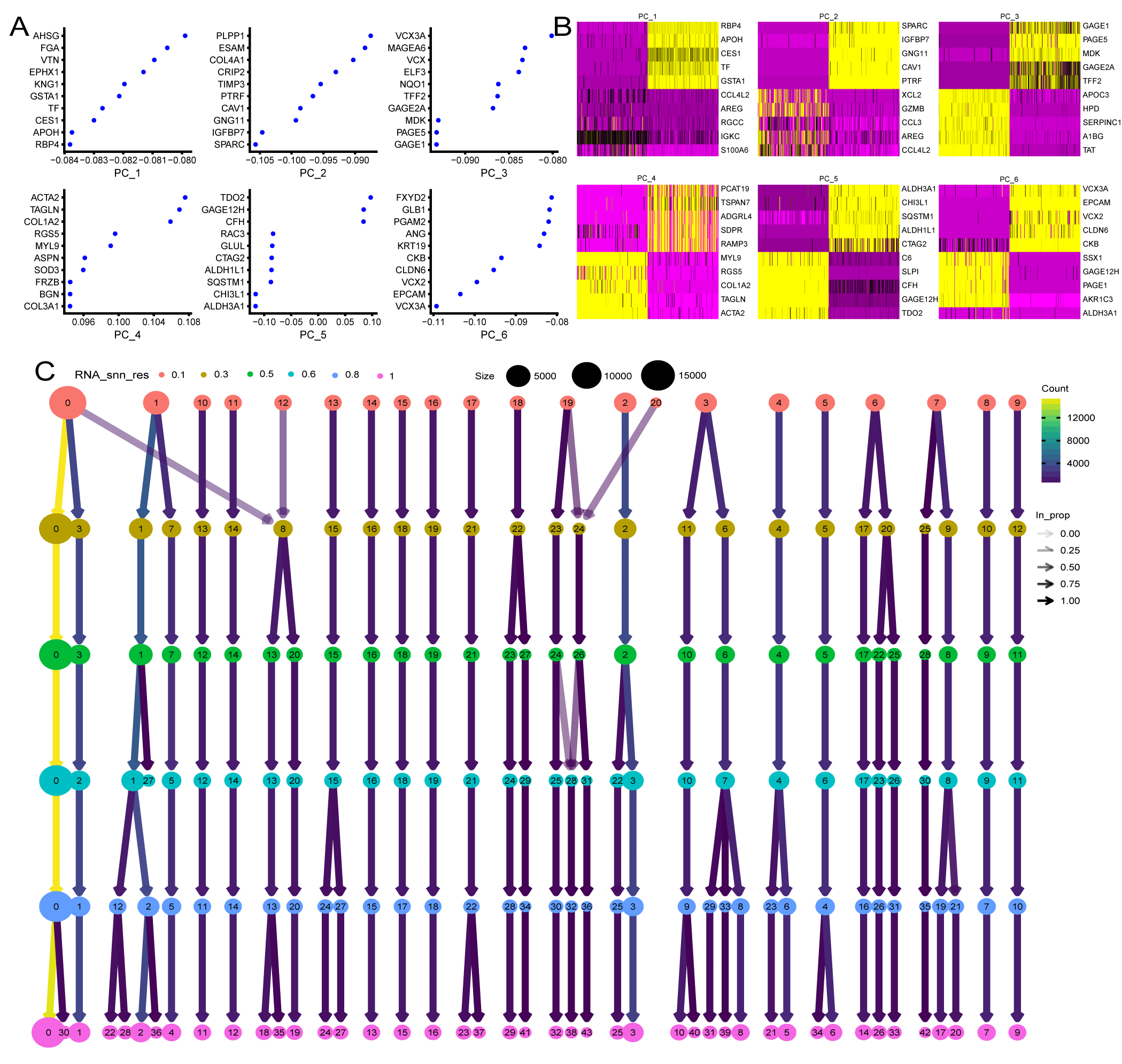


**Supplementary Figure 2: The significantly correlated genes in the top 6 PCs and the optimal resolution identification.** (A) The related genes of each principal component in the top 6 PCs. (B) Heat map showing the related gene expression in the top 6 PCs. (C) Sankey diagram displaying the cell clustering at 6 different resolutions (resolution = 0.1, 0.3, 0.5, 0.6, 0.8, 1).

**Supplementary Figure.3**


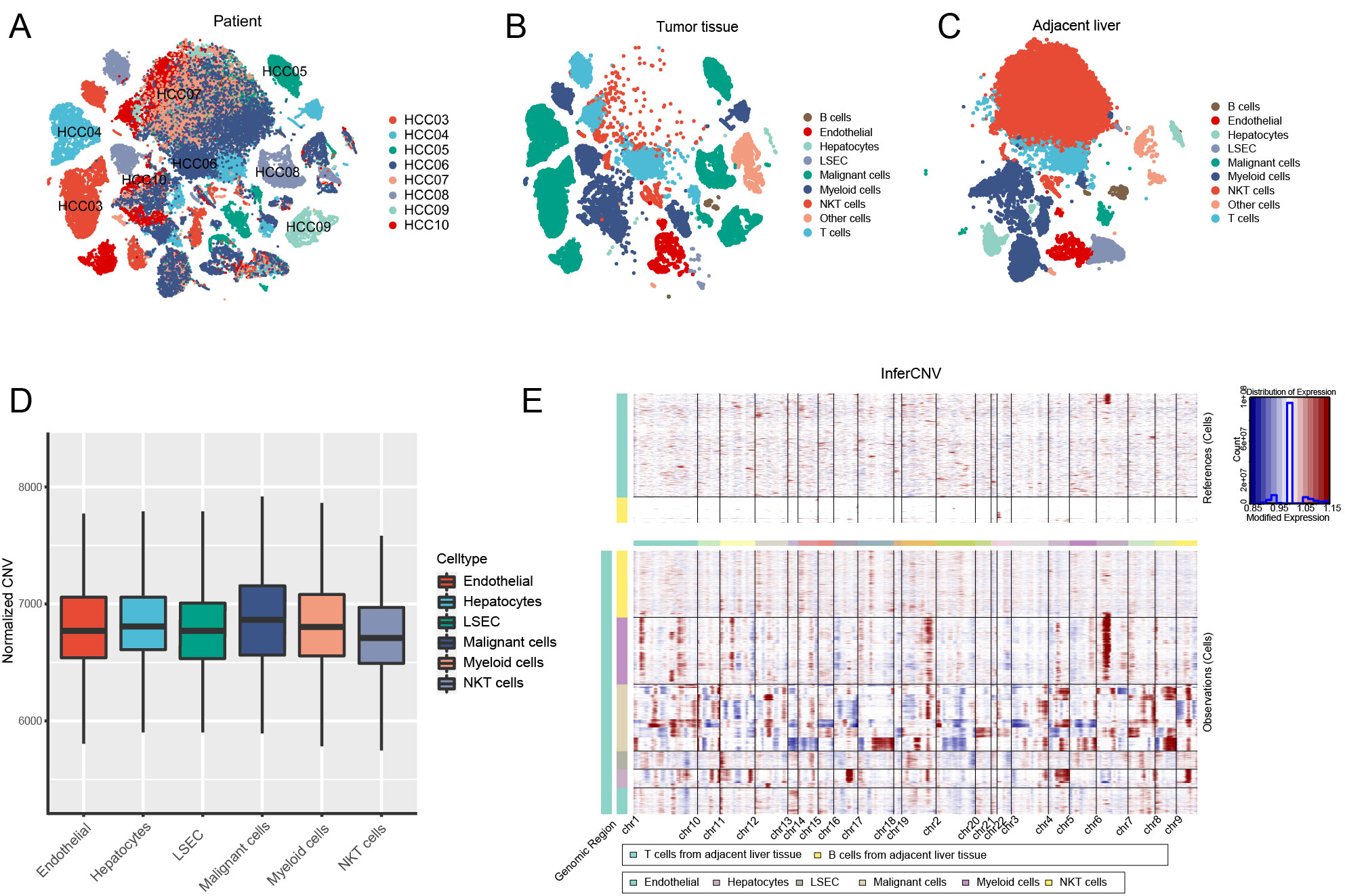


**Supplementary Figure 3: t-SNE plot from 8 HCC patients and CNV inference of mainly cell type.** The t-SNE plot showing patient origins (A) and cell cluster in tumor (B) and adjacent liver samples (C) by color. (D) The box plot showing the normalized CNV for different cell types. (E) The hierarchical heatmap showing large-scale CNV in various cell types.

**Supplementary Figure.4**


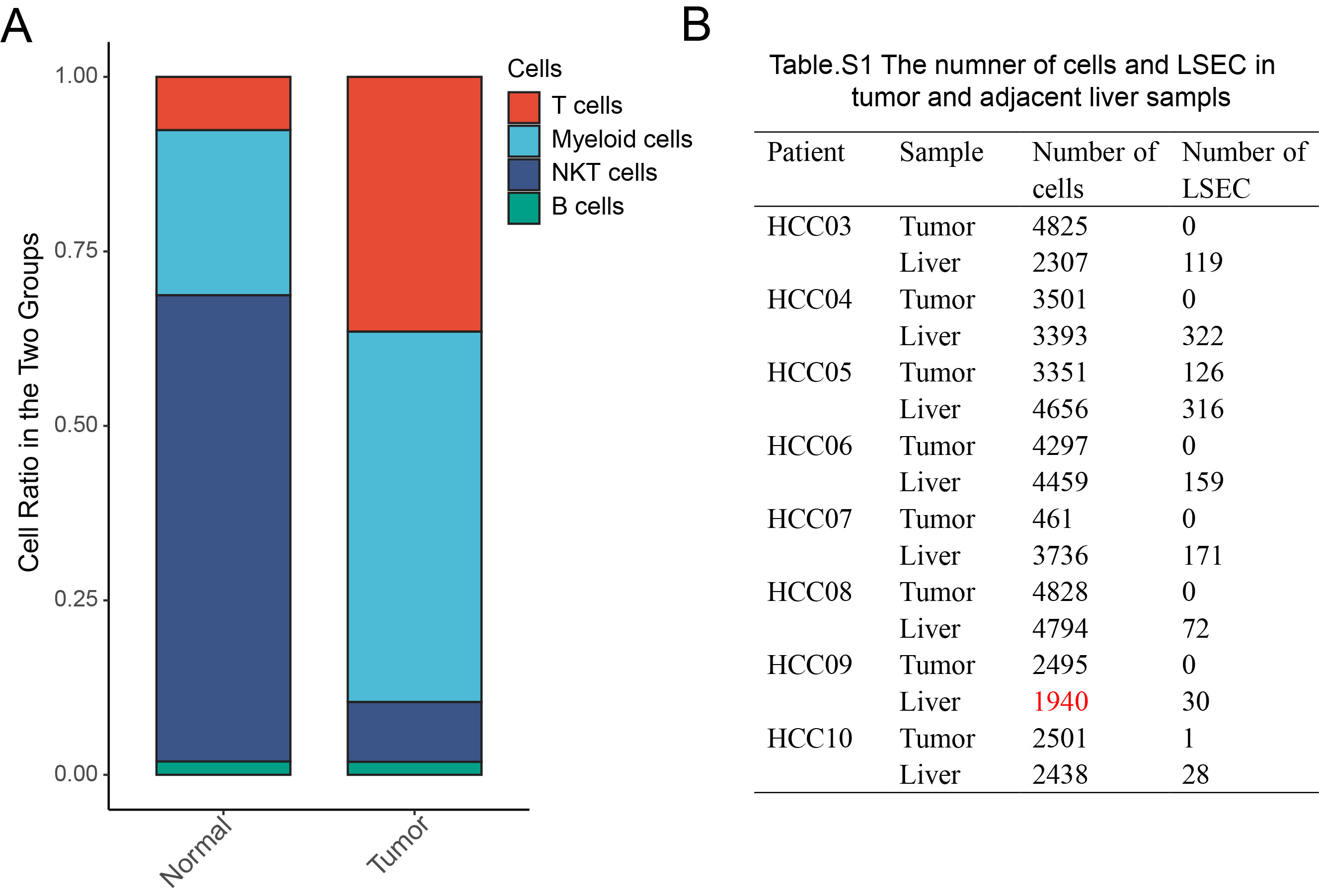


**Supplementary Figure 4:** (A) The histogram indicating the proportion of immune cells in normal liver and tumor tissue. (B) The table showing number of cells and LSEC on tumor and adjacent liver tissues for each patient.

**Supplementary Figure.5**


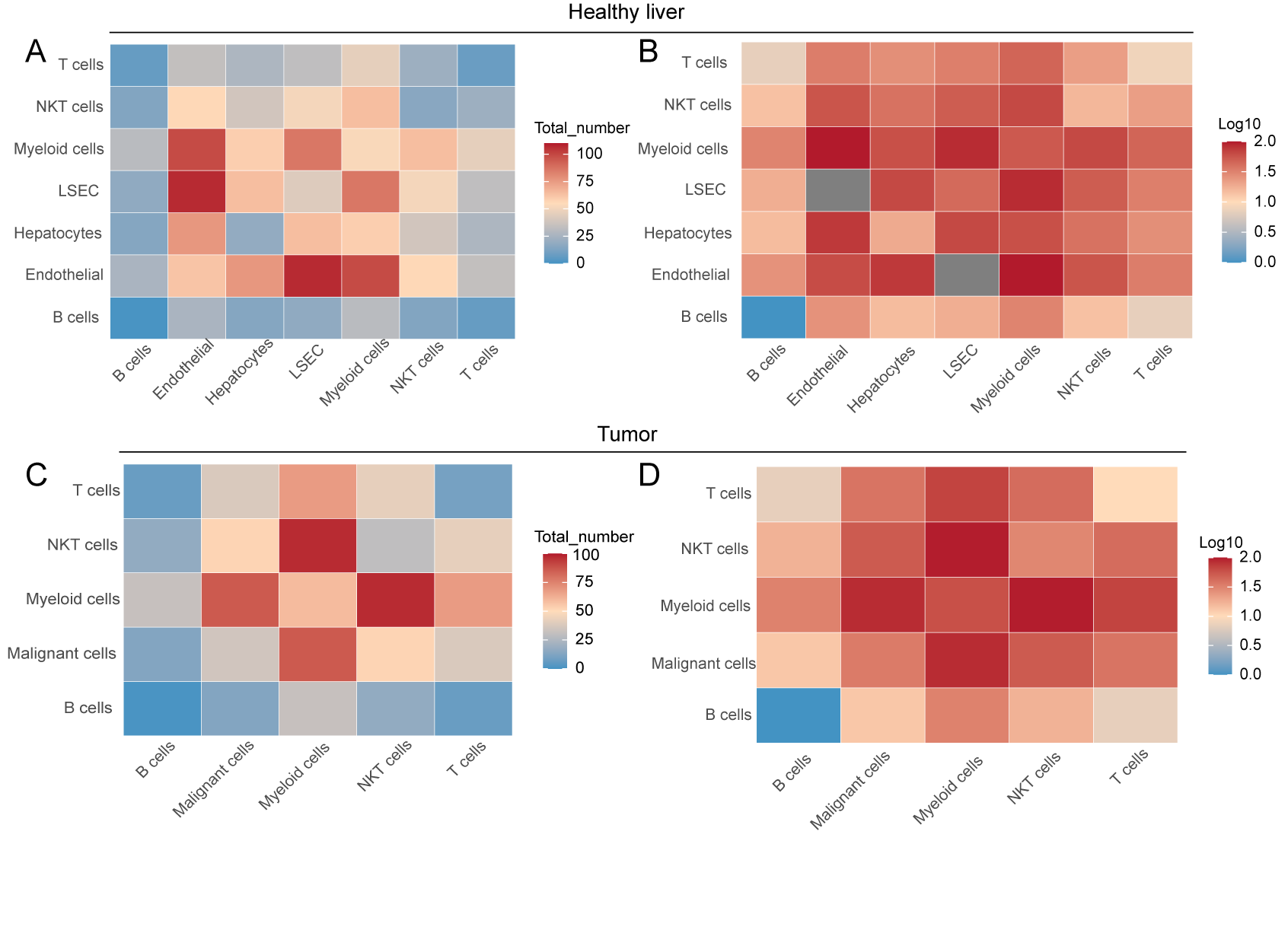


**Supplementary Figure 5: The heatmap showing the level of cell-cell interactions between each cell type and others in tumor and liver samples.** (A) The count value of putative ligand–receptor pairs between each cell and others in the healthy liver. (B) ligand–receptor pairs under log-count value. (C-D) The count value and log-count value of putative ligand–receptor pairs between each cell and others in tumor. Color key from blue to red indicates relative expression levels from low to high.

**Supplementary Figure.6**


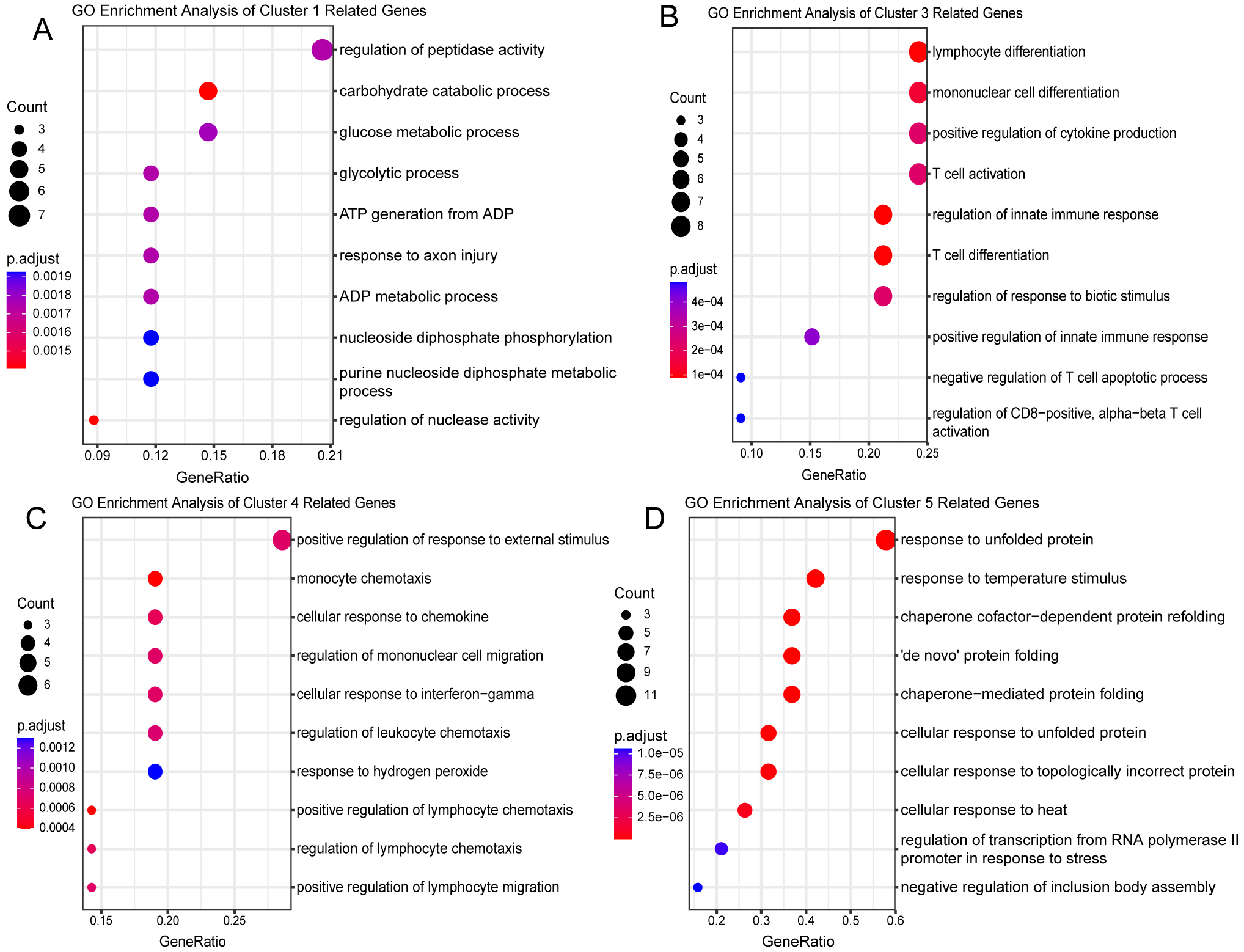


**Supplementary Figure 6: The GO enrichment analysis for biological processes of 149 dynamical genes with 4 clusters.** The Go functional enrichment analysis of cluster1 related genes (A), cluster3 related genes (B), cluster4 related genes (C) and cluster5 related genes (D)**.**

**Supplementary Figure.7**


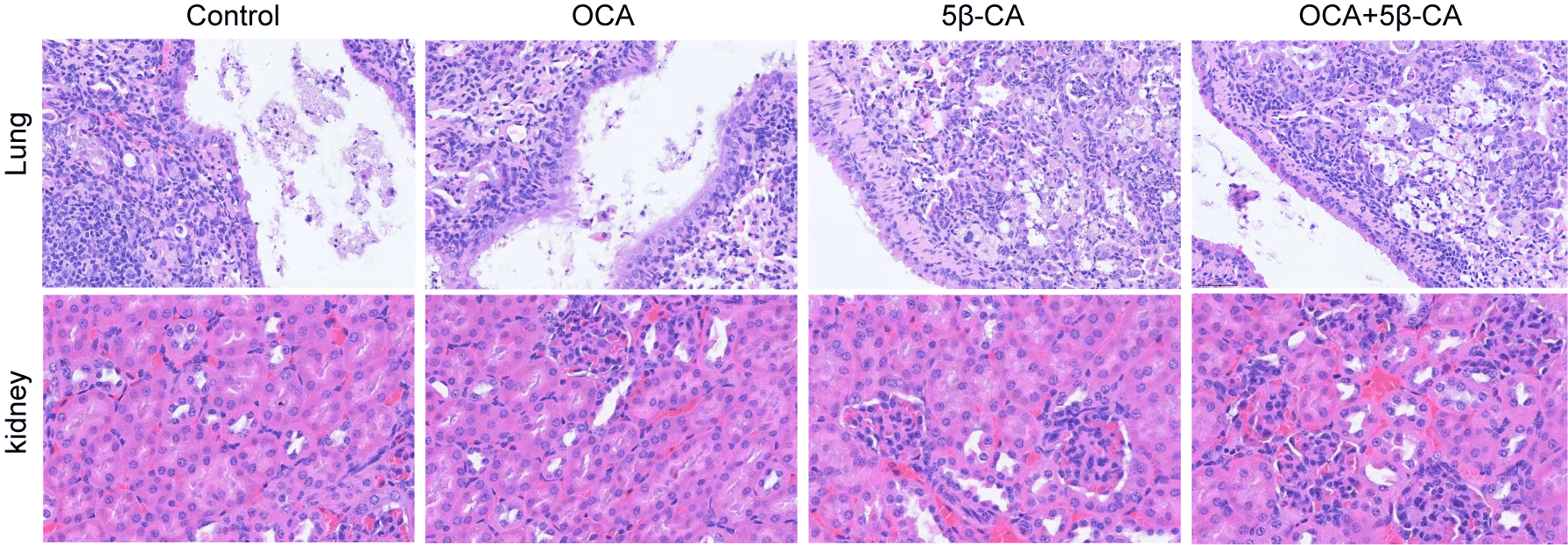


**Supplementary Figure 7: The H&E images for lung and kidney of mice treated with various drugs.** To assess the possible side effects of OCA and 5β-CA, the lung and kidney of mice were sampled for histological examination, and no obvious damage was observed in all groups.
